# ST-Segment Elevation Myocardial Infarction From Carney Complex Familial Myxoma Embolization

**DOI:** 10.1016/j.jaccas.2025.106120

**Published:** 2025-11-19

**Authors:** Ahad Firoz, Spencer Kitchin, Alirameen Akram, Saul Schaefer

**Affiliations:** a Department of Internal Medicine, University of California–Davis Medical Center, Sacramento, California, USA; b Division of Cardiovascular Medicine, Department of Internal Medicine, University of California–Davis Medical Center, Sacramento, California, USA

**Keywords:** chest pain, coronary angiography, left-sided catheterization, myocardial infarction, myocardial revascularization, palpitations, percutaneous coronary intervention, thrombosis, thrombus, valve repair

## Abstract

**Background:**

Carney complex (CNC) is a rare condition associated with multiorgan system tumors. Cardiac myxomas are a frequent complication of this disease.

**Case Summary:**

A 20-year old man with CNC presented with 3 days of dyspnea. Echocardiography revealed a rapidly growing left atrial myxoma. He developed chest pain (score: 10/10) during his hospitalization and was found to have an acute inferior ST-segment elevation myocardial infarction (STEMI). Emergent percutaneous balloon angioplasty and aspiration thrombectomy were performed, with eventual surgical myxoma excision.

**Discussion:**

STEMI due to myxoma embolization is rare and poorly understood. This case highlights the pathophysiology and complications associated with CNC, the acute therapeutic interventions for STEMI from myxoma embolization, and the long-term management of atrial myxomas.

**Take-Home Messages:**

In young patients with CNC, STEMI is often embolic from cardiac myxomas; acute treatment typically involves percutaneous coronary intervention with thrombectomy rather than stent placement. Definitive treatment involves surgical excision of the myxoma using a multidisciplinary approach, although recurrence rates are high in CNC.

## Visual Summary

**Figure:**
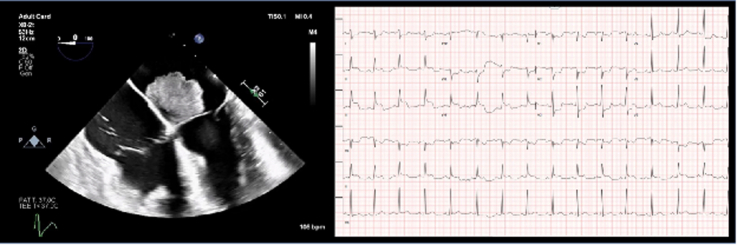

**Visual Summary:**
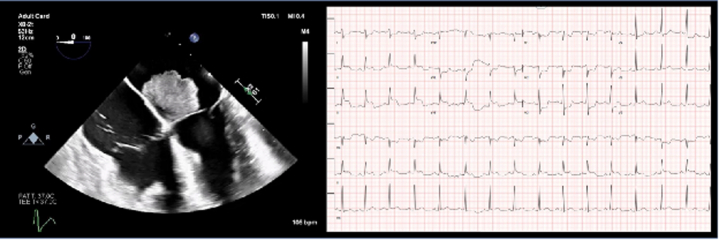
STEMI Due to Atrial Myxoma Embolization STEMI = ST-segment elevation myocardial infarction.

## History of Presentation

We present the case of a 20-year old man with a history of Carney complex (CNC) who arrived with 3 days of exertional dyspnea and palpitations. On examination, he was tachycardic with an additional diastolic heart sound consistent with tumor plop.

He was admitted for further evaluation with repeat echocardiography, and empirical antibiotics were started for suspected community-acquired pneumonia. Two days after presentation, he developed acute-onset severe chest pain (pain score: 10/10), somnolence, and significant hypotension.

**Take-Home Messages**

• In young patients with Carney complex, STEMI is often embolic from cardiac myxomas; acute treatment typically involves percutaneous coronary intervention with thrombectomy rather than stent placement.
• Definitive treatment involves myxoma surgical excision using a multidisciplinary approach, although recurrence rates are high in Carney complex.

## Past Medical History

The patient's pertinent medical history was notable for CNC; atrial masses; and Cushing syndrome for which he had undergone bilateral adrenalectomies and was on fludrocortisone and hydrocortisone.

**Figure 1:**
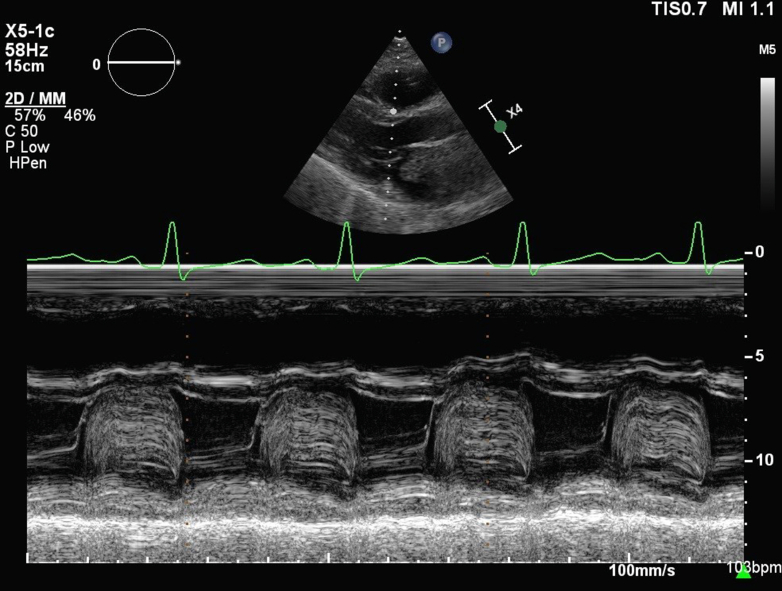
Transthoracic M-Mode Tracing of the Mitral Valve

**Figure 2:**
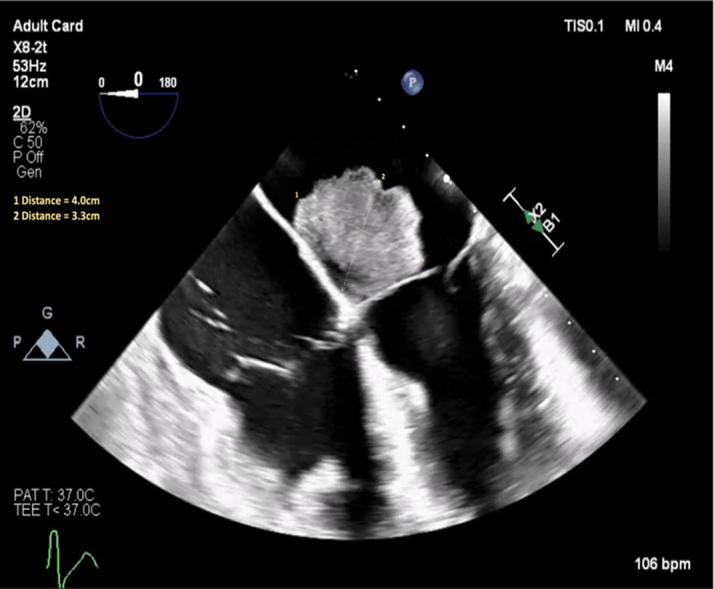
Transesophageal Echocardiography With Multilobulated Atrial Mass

**Figure 3:**
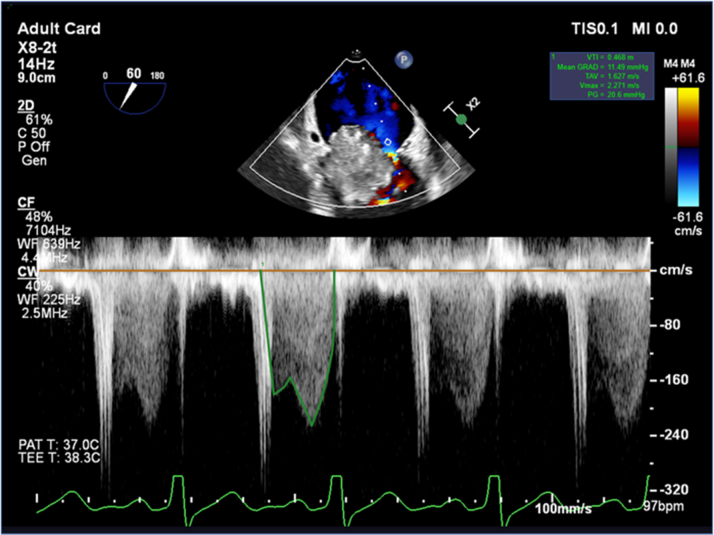
Transesophageal Mean Pressure Gradient of the Mitral Valve

Serial echocardiography demonstrated rapid interval progression of his atrial masses. Transthoracic echocardiography in May 2025 revealed a 2.0-cm, round, mobile mass at the base of the mitral valve that was most consistent with myxoma; transthoracic M-mode tracings of the mitral valve are included in Figure 1. Two months later, a transesophageal echocardiogram (Figure 2) showed a multilobulated mass attached to the inferobasal portion of the interatrial septum near the crux, extending across the aortomitral curtain with significant diastolic prolapse into the mitral valve (MV). This resulted in obstructive physiology, with a mean MV gradient of 11 mm Hg at a heart rate of 97 beats/min (Figure 3). Of note, the mass had enlarged to 4.0 × 3.3 cm. Additionally, a smaller (0.5 × 0.5 cm), circumferential mass was identified at the fossa ovalis on the left atrial side.

## Differential Diagnoses

Considerations included septic shock, cardiogenic shock, myocardial infarction, pulmonary embolism, and adrenal crisis.

## Investigation

**Figure 4:**
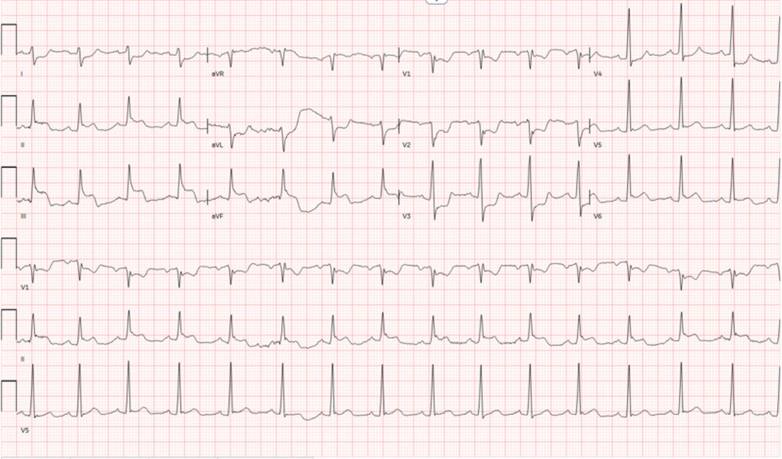
Electrocardiography Demonstrating Inferior STEMI STEMI = ST-segment elevation myocardial infarction.

**Figure 5:**
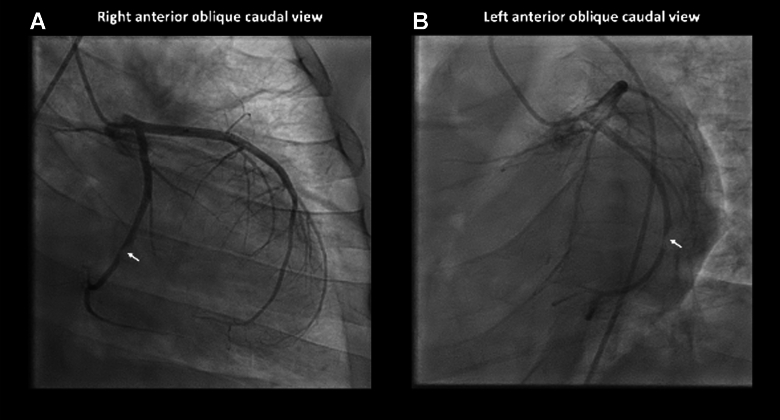
Coronary Angiography Illustrating Subtotal Occlusion of the Mid Left Circumflex Artery (A) Right anterior oblique and (B) left anterior oblique caudal views.

**Figure 6:**
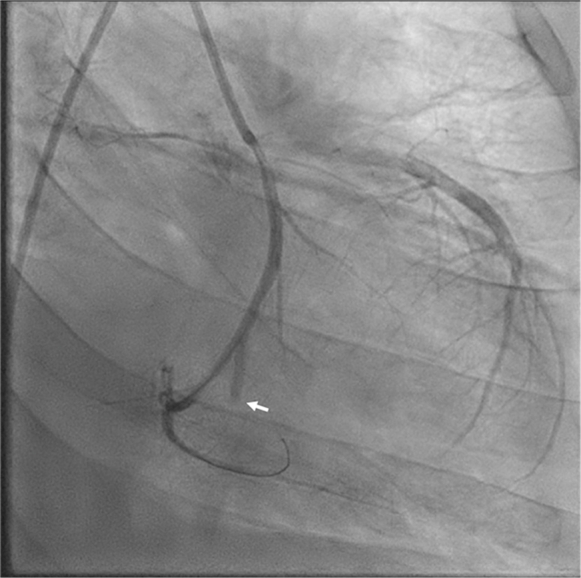
Coronary Angiography (Left Anterior Oblique Caudal View) After Aspiration Thrombectomy Angiography revealed a large, acute occlusion of the third obtuse marginal branch.

Electrocardiogram revealed inferior ST-segment elevation myocardial infarction (STEMI), as demonstrated in Figure 4. The patient was transferred emergently to the cardiac catheterization laboratory where he was intubated due to somnolence. Coronary angiography revealed subtotal occlusion of the mid left circumflex artery (LCx) and total occlusion of the third obtuse marginal branch (OM3) (Figures 5 and 6).

## Management and Outcome

**Figure 7:**
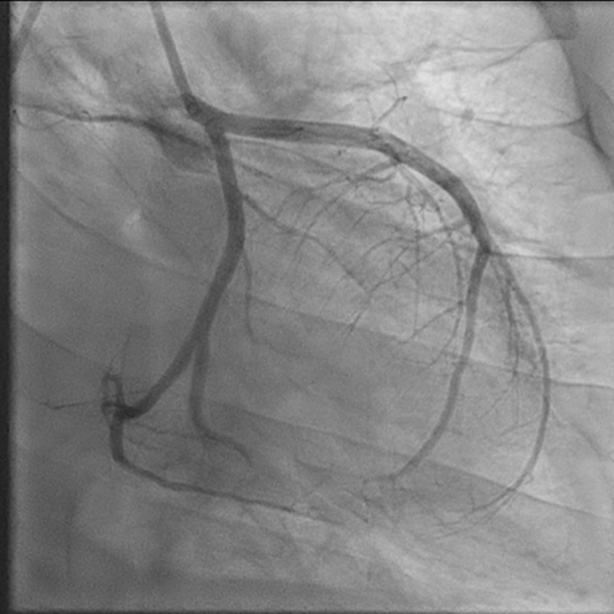
Coronary Angiography (Left Anterior Oblique Caudal View) After Percutaneous Coronary Intervention

Percutaneous coronary intervention (PCI) with aspiration thrombectomy was performed at the mid-LCx lesion, yielding several small thrombi. Repeat cineangiography revealed acute occlusion to OM3, which was intervened upon with percutaneous balloon angioplasty. Postintervention angiography (Figure 7) demonstrated TIMI flow grade 3 down the LCx, with no residual embolus, and TIMI flow grade 2 in the OM3, with possible distal thrombus burden. Given persistent hypotension during the procedure, an intra-aortic balloon pump was placed. The patient was admitted to the intensive care unit for cardiogenic shock, requiring continued vasopressor and inotropic support. Computed tomography scans of his chest and head did not reveal evidence of further embolic phenomenon. Repeat transthoracic echocardiography showed preserved ejection fraction, with significant mitral inflow obstruction resulting in mitral stenosis. After a few days, his cardiogenic shock improved, allowing for removal of the intra-aortic balloon pump.

**Figure 8:**
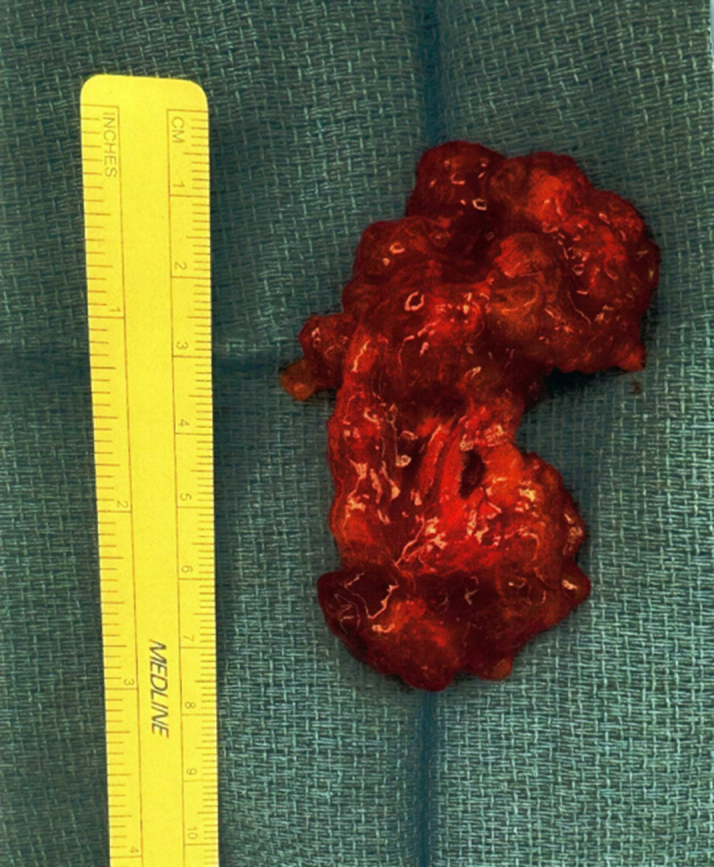
Excised Atrial Myxoma

The cardiac surgery team was consulted for atrial mass excision. Given the patient's comorbidities, a multidisciplinary team approach was used, including specialists from cardiovascular medicine and surgery, pulmonology, cardiothoracic critical care medicine, endocrinology, and neurologic surgery. The left atrial mass was successfully excised; media images are included in Figure 8. Pathology confirmed the diagnosis of atrial myxoma. Intraoperatively, rupture of the anterior middle mitral leaflet (A2) with moderate to severe regurgitation was identified, prompting MV repair with a 34-mm 4DMemo Plus annuloplasty ring (Corcym). Further, left atrial appendage closure was performed with a 40-mm Penditure clip (Medtronic). Overall, the patient tolerated the procedure well.

His postoperative course was complicated by atrial flutter requiring amiodarone, which precipitated tachy-brady syndrome, as well as new-onset heart failure with transient decline in ejection fraction to 40%, later improving to 55%. The patient was discharged with guideline-directed medical therapy agents, with plans to continue optimization in the outpatient setting.

## Discussion

Carney complex is a rare clinical syndrome associated with mutations in the *PRKAR1A* gene, which codes for a protein kinase A regulatory subunit.^1^ Although the majority of patients acquire CNC in an autosomal-dominant fashion, an estimated 25% of cases arise sporadically as de novo mutations.^1^ The disease process is characterized by the tetrad of pigmented skin lesions, endocrine tumors, nonendocrine tumors, and myxomas.^1^^,^^2^ Of these conditions, cardiac myxomas affect 52% of CNC patients and are the second most frequent manifestation of the disease; additionally, cardiac myxomas are the most common cause of mortality and morbidity for this population.^2^

Although atrial myxomas are benign tumors, they have the potential for serious complications, such as valvular obstruction and embolization. In the instance of valvular obstruction, patients may present with syncope, heart failure, or pulmonary hypertension, as observed in the current case. Additionally, embolic phenomenon is not uncommon in untreated myxomas, with some studies estimating myxoma embolization occurrence rates reaching as high as 20%.^3^ Risk factors for embolization include atypical location, irregular appearance, and elevated platelet volume and count.^3^ For left atrial myxomas, the most common location for embolization is the central nervous system; embolization to the coronary system is rarely observed, likely because of the acute takeoff of the coronary vessels from the aortic root compared with the relatively linear path of the central nervous system and systemic circulation from the left ventricular outflow tract.^4^^,^^5^

Coronary artery embolisms (CEs) have been reported to account for approximately 4.3% of all STEMI cases; of this fraction, myxomas were responsible for 1.9% of CEs; other more frequent etiologies of CE include atrial fibrillation (28%), malignancy (15%), and dilated cardiomyopathy (9%).^6^ Case reports of embolic cardiac myxomas causing STEMI are rare in the literature, with limited information available on diagnostics and management. In the current case, our patient presented with anginal chest pain and was found to have STEMI as a result of myxoma embolization. Diagnostic clues to the underlying etiology of his cardiac event include his young age, lack of atherogenic coronary artery disease, presence of multiple acute coronary artery lesions, and large left-sided atrial myxoma. In less clear clinical cases, Shibata et al's^7^ definition for CE, consisting of 3 major and 3 minor criteria, has been widely accepted in the medical community; using this methodology, our patient's score was “definite CE.”

The acute management of CE resulting in STEMI involves emergent coronary angiography. Distal or small emboli may be managed conservatively with intravenous antithrombotic agents, whereas larger thrombi often require PCI.^8^ Although there are no clear guidelines on choice of agents, unfractionated heparin, glycoprotein IIb/IIIa inhibitors, or bivalirudin have previously been suggested.^5^ Occasionally, wire manipulation may sufficiently open occlusions to allow for intrinsic thrombolysis; however, in cases of large thrombus burden in a proximal vessel, thrombectomy may be necessary.^8^ Given the pathophysiology of the embolic process, stent placement is rarely required. After PCI, definitive therapy involves surgical removal of the myxoma. Given the systemic disease process of CNC, a multidisciplinary approach is crucial to optimize patient outcomes.

Although surgical excision of myxoma is frequently curative in the general population, patients with CNC have a high rate of myxoma recurrence. In a retrospective study investigating cardiac myxomas in CNC patients, the investigators found that the first, second, third, and fourth recurrence rate of cardiac myxomas were 44%, 35%, 38%, and 50%, respectively, of each sequential myxoma cohort.^9^ In another study, the authors reported that 28% of CNC patients with cardiac myxomas required ≥2 operations because of myxoma recurrence. As such, CNC patients are at high risk for repeat sternotomies and the additional inherent intraoperative complexities given scar tissue deposition and adhesions; under certain conditions, as an alternative for patients with an extensive history of prior sternotomies, a thoracotomy approach may be considered.^10^ Given the high myxoma recurrence rate, regular monitoring through serial echocardiography is crucial for CNC patients. While there are no specific guidelines for familial myxoma surveillance, annual echocardiography screening is generally recommended, with screening every 6 months for those with cardiac myxoma(s).^1^

## Conclusions

Our case report describes the rare presentation of a young man with CNC who experienced coronary artery embolization from familial cardiac myxoma, resulting in inferior STEMI. Embolization should be suspected in young patients with little to no coronary artery disease, multivessel coronary involvement, and a clear embolic source. The patient underwent emergent aspiration thrombectomy and balloon angioplasty; once he was stabilized, the myxoma was surgically excised. Despite surgical removal, patients with CNC have a high rate of cardiac myxoma recurrence, which warrants surveillance echocardiography every 6 months to 1 year.

## Funding Support and Author Disclosures

The authors have reported that they have no relationships relevant to the contents of this paper to disclose.
